# Engaging children, young people, parents and health professionals in interviews: Using an interactive ranking exercise within the co-design of multimedia websites

**DOI:** 10.1177/13674935221109684

**Published:** 2022-06-24

**Authors:** Jacqueline Martin-Kerry, Steven Higgins, Peter Knapp, Kristin Liabo, Bridget Young

**Affiliations:** 1 School of Allied Health Professions, 4488 University of Leicester, Leicester, UK; 2 School of Education, 3057 University of Durham, Durham, UK; 3 Department of Health Sciences and Hull York Medical School, 8748 University of York, York, UK; 4 NIHR Applied Research Collaboration South West Peninsula, 171002 University of Exeter Medical School, Exeter, UK; 5 Department of Public Health, Policy and Systems, Institute of Population Health, 4591 University of Liverpool, Liverpool, UK

**Keywords:** child, adolescent, parents, health professional, qualitative research

## Abstract

When planning paediatric trials, it is important to consider how best to communicate with children and young people (CYP) so they understand what they are taking part in. It is also important to consider what information they need. Involving CYP as research participants leads to research that is more relevant although it can be difficult to engage CYP in qualitative research to improve trial materials due to the topic area. This paper describes how a visual ranking exercise within qualitative interviews acted as a helpful conduit to engaging discussions to inform a co-designed website with information for trial participants. 40 people participated in interviews during which the ranking exercise was used (11 CYP aged 9–18 years, 14 parents, 15 professionals). We found the ranking exercise supported participant engagement and prevented them feeling that particular responses were expected. It also enabled participants to discuss their ranking (and decisions behind this) with other participants and the researcher. Co-design interviews with CYP that use interactive exercises such as ranking are likely to elicit richer data than those relying on traditional questioning techniques.

## Introduction

It is internationally recognised that paediatric trials are important to inform healthcare decisions (Joseph et al., 2015; Klassen et al., 2008). However, paediatric trial recruitment is difficult (Tishler and Reiss, 2011), with the need to consider both the child and parent’s perspectives about taking part (Caldwell et al., 2004) and that the child’s capacity to understand what a trial entails varies according to their developmental level and maturity (Nuffield Council on Bioethics, 2015). Risks of trial participation for children and young people (CYP) such as discomfort or distress from procedures may also be different compared to adults considering participation (Caldwell et al., 2004). Ethical guidance is clear that while CYP might have limited capacity, they should have opportunities to understand what any research would entail, and participate in the decision-making process (Nuffield Council on Bioethics, 2015). Printed participant information about trials is usually provided to potential participants and these documents should be understandable to assist decision-making. However, participant information sheets for paediatric clinical trials are often lengthy and difficult to read and understand (Caldwell et al., 2012; Crane and Broome, 2017) with implications for CYP’s understanding of trials, which may affect their decision to participate.

Involving CYP in designing and prioritising research is important to ensure that it is appropriate and relevant to them (Ford et al., 2007; Martin-Kerry et al., 2019). CYP can provide important insights into their lived experiences and their preferences for receiving information about trials (Curtin, 2001; Patel et al., 2016). However, it may be difficult to engage CYP in qualitative research when topics are potentially difficult, technical or unfamiliar, such as discussing participating in a trial (Dunhill et al., 2009). A recent systematic review reported different data collection methods for research involving CYP including the use of digital technologies, qualitative interviews, audio diaries and art (Flanagan et al., 2015). Other interactive techniques to engage CYP have been described (Clark, 2005; Flanagan et al., 2015; Lobinger and Brantner, 2020; Morgan et al., 2002; Thomas and O’Kane, 1998). These include using visual aids such as photographs and cards, cartoon templates (Wall and Higgins, 2006) and ranking exercises such as the ‘Diamond Ranking Exercise’ (Thomas and O’Kane, 1998). The review by Flanagan et al. was on data collection and did not include methods where participants can rank or order information (Flanagan et al., 2015). Whilst publications refer to the benefits of using interactive visual methods with CYP in health research, it is rare to find detailed descriptions of how these methods work in practice and assist researchers (Christian et al., 2010; Fargas-Malet et al., 2010; Gibson et al., 2010; Kirk, 2007; O’Connell, 2013; Punch, 2002; Vindrola-Padros et al., 2016).

## Aim

To explore whether a visual ranking exercise is useful for CYP in identifying which information they feel is important to know before taking part in a clinical trial.

## Methods

The larger TRials Engagement in Children and Adolescents (TRECA) study (Martin-Kerry et al., 2017) was co-designing multimedia websites for paediatric trials as an alternative to traditional printed information sheets. Before we (the study research team) could create the websites we needed to understand stakeholder preferences for the websites in terms of content and appearance. We did this by interviewing CYP, parents/carers and professionals.

### Interview process and structure

This co-design study required CYP to engage with concepts about trials during the interviews, and we anticipated that this would be difficult for CYP. We designed an interactive information ranking exercise to assist their engagement. The exercise aimed to help them discuss what they would want and need to see on a multimedia website about a paediatric trial. Although our focus was on engaging CYP during the interviews, we also wanted to include other key stakeholders including parents and professionals involved in paediatric trials and seek their thoughts and rankings of these topics. We used the ranking exercise with all participants. The websites were user-tested (Sheridan et al., 2019) ahead of their use in paediatric trials. We undertook two rounds of interviews (individual, joint or focus groups). The first round focused on participants’ experiences in research, and what information they considered important before making a decision about participation in a paediatric trial. The second round sought their views of draft multimedia websites developed based on data collected in the first round of interviews. This paper focused on the specific ranking exercise method used within the first round of interviews.

To develop the multimedia websites, we needed to prioritise which pieces of information to include on the websites to ensure they were functional but also engaging. Those approached about a trial are often faced with large amounts of information, and we wanted to find ways to present this effectively so that people can find key information easily and avoid presenting them with text-heavy webpages. Our plan was to identify information during the interviews that was most important for those approached about a trial to know before making a decision. This information was then more prominent on the websites but we would also include other information, deemed by participants as less important, in less prominent areas of the websites. We anticipated that a ranking exercise would enable the various pieces of information to be prioritised. Importantly, we wanted to make the ranking fun and engaging for CYP, and facilitate discussion.

We identified 20 pieces of information for ranking, based on a systematic review about the information adults needed before deciding whether to participate in a trial (Kirkby et al., 2012). We reviewed existing ranking methods and identified the diamonds exercise (Rockett and Percival, 2002; Thomas and O’Kane, 1998) as promising. However, this needed adaptation for our purpose as it had a maximum number of topics (usually nine), whereas we needed to include 20 pieces of information. We therefore used a ranking exercise that involved an A0 size mock target and laminated brightly coloured cards which named each topic. The activity combined the ‘ranking’ and ‘sorting’ features recommended by Colucci (2007), with participants being asked to place the cards on different areas of the target according to its importance (Colucci, 2007). Topics on the cards are listed in Table 1.

**Table 1.** Topics that were used on cards (this wording was simplified for interviews with children).

• Why is the study happening?
• Why have I been invited?
• Do I have to take part?
• What will happen if I don’t want to carry on with the study?
• Will it cost me any money to take part?
• What will happen to me if a take part?
• Will I be paid for taking part?
• What is being tested in the study (for example, what drug or device)?
• What are the different treatments or types of healthcare being provided in the study?
• What are the risks of taking part?
• What are the possible benefits of taking part?
• What happens when the study ends?
• What would happen if a problem happens in the study/could I make a complaint?
• Will my taking part be kept confidential (private)?
• Will my GP/family doctor be told about my taking part?
• What will happen to any blood tests or other samples that I have as part of the study?
• What will happen to the results of the study?
• Who is running the research?
• Who is paying for the research?
• Who has reviewed the study?

### Sampling

We recruited CYP with a long-term health condition, as well as their parents/carers, and professionals involved in paediatric research. Inclusion criteria for CYP were that they must be aged between 6–18 years of age with a long-term health condition. Parent/carer participants must have a child under 18 years of age with a long-term condition; professionals must have experience of recruiting CYP to a trial. Participants who could not provide informed consent/assent or required an interpreter were not included in this study. For CYP and parents/carers, we recruited through two routes: a children’s hospital in North West England and a Generation R Young Persons’ Advisory Group (YPAG) located in the Midlands, comprising CYP who advise on the design of research involving CYP in the NHS. Recruitment via the hospital route involved nurses contacting, either by phone or in person, families of CYP who were receiving specialised care for long-term conditions; the YPAG route involved the coordinator contacting members and their parents by text message or phone call. JMK telephoned those who agreed to contact to explain the study and arrange a time for an interview. Sampling of CYP and parents aimed to encompass variation in age, gender, long-term health condition, trial experience and ethnicity. Professionals were those involved in paediatric research, such as paediatricians, research nurses and data managers. They were initially emailed by an academic paediatrician working in the study hospital. Those who expressed an interest were contacted by JMK by email to arrange an interview. Sampling of professionals aimed for diversity in roles and paediatric specialities within trial settings.

### Data collection

JMK, trained and experienced in qualitative research, conducted topic-guided, semi-structured interviews. These were undertaken as either individual interviews, joint interviews, or focus groups. All round one interviews (those with CYP, parents/carers, professionals) were conducted July–October 2016. Participants were asked to participate in focus groups but could choose to instead participate in joint interviews (e.g. parent/carer and child) or individual interviews. Interviews began narratively with JMK asking participants about their experiences in research, and more specifically in trials. JMK then introduced a ranking exercise and asked participants to rank topics as ‘important’ (yellow; centre of target), ‘somewhat important’ (red) or ‘not important’ (blue) by attaching cards to the relevant area of the target. JMK undertook this activity with all participants – CYP aged 9–18 years, parents/carers and professionals. After the exercise, JMK asked participants about their needs and preferences for the presentation of information on websites and used examples of websites and designs for discussion. During interviews JMK made observational notes describing participants’ interactions and behaviours during the interviews, and she extended these notes immediately after the interviews. All interviews were audio-recorded and transcribed; transcripts were checked and pseudo-anonymised before analysis.

### Data analysis

Analysis drew upon thematic approaches (Braun and Clarke, 2006, 2014). This involved reading interview notes and transcripts multiple times to aid familiarisation and to identify recurring patterns of interaction during the ranking exercise, then organising these into themes. Particular attention was paid to the observational notes describing how participants interacted during the ranking exercise, and linking these to transcribed data on what participants had said. We also compared observational and transcribed data across participant groups to identify similarities and differences. Analysis was undertaken by JMK with regular meetings with BY to discuss transcripts and aid data interpretation. Microsoft Excel and Word were used to assist data management (La Pelle, 2004).

### Ethics

Yorkshire and the Humber Research Ethics Committee (16/YH/0158), the Health Research Authority (IRAS ID, 195,396), and the various NHS Trust Research and Development Departments involved, approved the study. All participants were provided with an age-appropriate printed participant information sheet (PIS) and signed an assent or consent form. Participants received a £10 voucher to thank them for their participation and time. Travel costs were covered.

## Results

### Participants

Forty people participated in round one interviews (11 CYP, 14 parents/carers, 15 professionals). CYP were aged 9–18 years and had a range of health conditions including rheumatoid arthritis, respiratory conditions (e.g. asthma), type 1 diabetes, and different forms of cancer (e.g. leukaemia). Four CYP attended a focus group; the remaining CYP were interviewed in joint interviews with one or both parents present. Nine parents/carers participated in joint interviews with their children; three parents were interviewed individually (one via Skype, the others face-to-face); and two parents participated in an interview together. The professionals were interviewed in three focus groups (two, four and nine participants per group).

### Findings

At the start of the interviews, participants talked about their experiences with research and clinical trials. Professionals talked about their experiences of approaching families about research, recalling the questions that they often asked. In talking about their experiences, CYP often recollected how they felt when approached, and the types of questions they had at the time. Some CYP had not been approached about participating in a trial but talked about what they thought they would want, or need, to know before deciding. Parents talked about their experiences of being involved in discussions about their child’s participation in trials or other research studies. Participants seemed comfortable talking about these experiences and preferences but tended to speak directly to the researcher rather than to each other, except for the professional focus groups. In their focus groups, it was clear that professional participants worked with each other and knew each other well, and they regularly added or responded to each other’s comments.

Through analysis of the transcripts and consideration of notes made immediately after each interview or focus group, two themes were identified as being benefits of the ranking exercise: ‘reflections on research participation’ and ‘engagement and enjoyment’.

### Reflections on research participation

Many CYP vividly recounted their experience of being invited to participate in a trial. For example, one CYP described her experience of being approached about an oncology trial a few years earlier:

“So it was a bit of a weird study because almost like a couple of days after diagnosis my mum was given a ridiculous amount of paperwork which wasn’t really timed particularly well, to say that you’d consider the trial, so that meant you weren’t even consenting to the trial but they needed you to consider the trial which I wasn’t really sure why and I’m still not sure why… and then so the existing protocol was two intensification blocks, like the treatment with a 6 month gap in between and they were considering cutting it down to only one because they thought the amount of medication given was excessive.” (CYP/18, female, 18 years)

**Figure 1.**
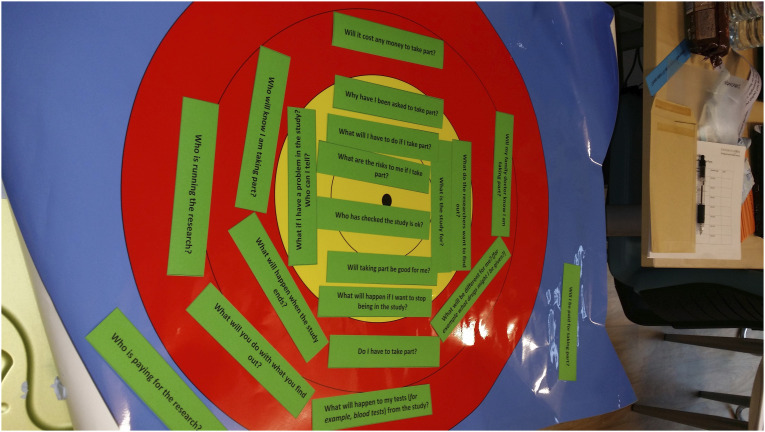
Activity undertaken by 10-year-old during interview.

Most CYP were engaged and interested in talking about their experiences of research during the more traditional part of the interview where the researcher asked questions. However, the energy within the room changed when JMK introduced the ranking exercise. Some CYP jumped out of their seat in excitement at the opportunity of doing an activity; often keen to be able to choose the colour of the laminated topic cards. They were often drawn to cards that were bright colours such as blue or green. When JMK explained how the exercise would work and that the placement of the cards would be decided by how important the participant thought the topic was, some CYP were very animated. For example, in one interview with a 10-year-old and his mother (see Figure 1):

interviewer: “….but what I want to find out from you is whether you would be able to put them in terms of what’s important to you. So if you think something is really important put it in the middle….”

“Yeah target, where you want the hit!” (CYP/16, male, 10 years)

**Figure 2.**
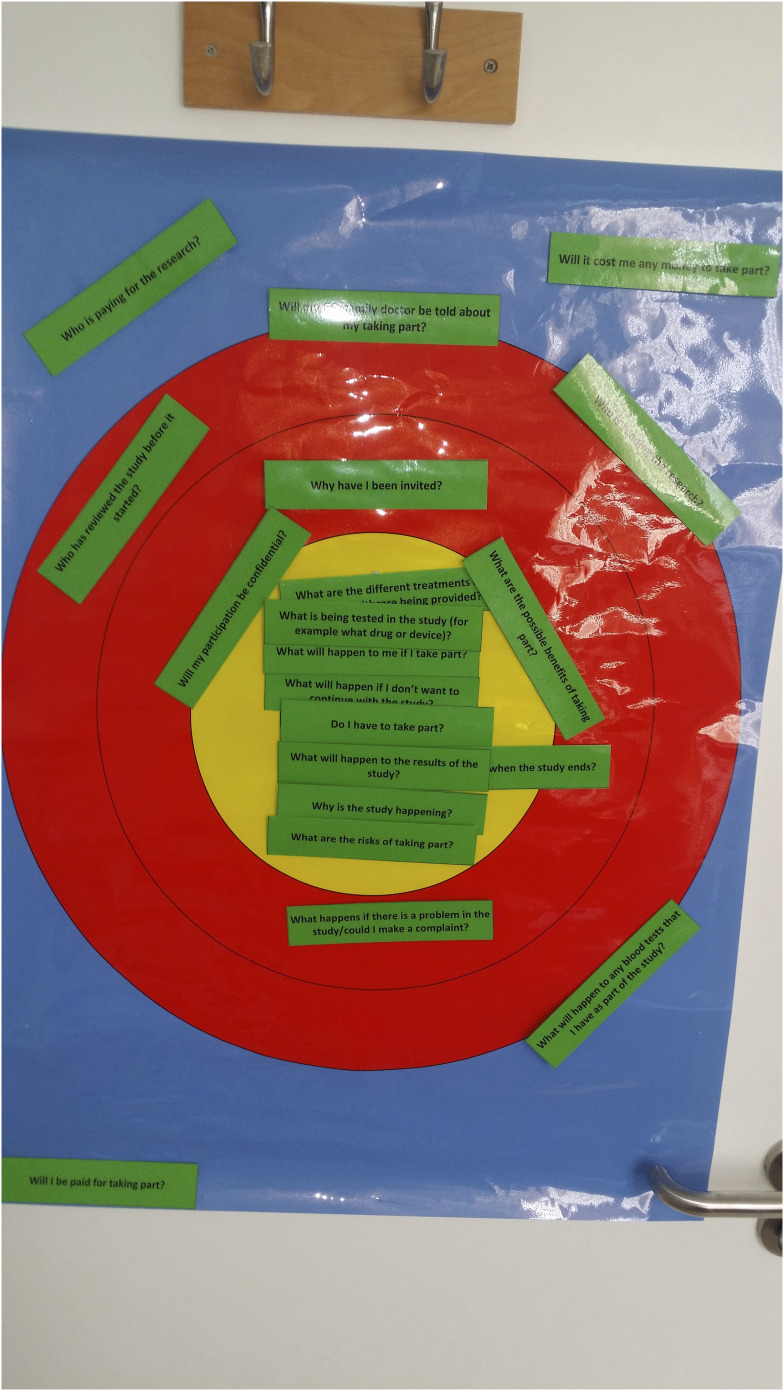
Activity undertaken by four young people aged 15–18 years in a focus group.

However, two CYP in a focus group initially looked hesitant when the ranking exercise was mentioned. They first looked at the floor, then briefly at each other. It seemed they were concerned that they would have to pro-actively contribute rather than respond to questions when they had something that they wanted to say. JMK encouraged participants to join in the exercise. Any concern about the exercise seemed to cease once the young people began ranking cards and they were actively chatting with each other as each card was picked out by one of the CYP. CYP would enthusiastically read out a card and ask others ‘what do you think’? They described the process as ‘fun’ and ‘interesting’ and challenged each other’s initial response about how important a topic was. They did this in a friendly manner, often with bursts of laughter or enthusiastic statements. This enabled them to discuss, as a group, the initial response and decide together about the final ranking (See Figure 2). For example, in the focus group of CYP discussing the ranking of the question cards was particularly interactive:

“‘Why have I been invited?” [reading out card] [Pause, laugh] (CYP/18, female, 18 years)

“I assume that’s to the research as in why they’ve selected you to actually participate, I suppose.” (CYP/20, male, 18 years)

“Isn’t it kind of obvious…explanatory?” (CYP/18 female, 18 years)

“I guess sometimes it wouldn’t be… especially in randomised trials, it’s not.” (CYP/20, male, 18 years)

“So I think, there’s like, there’s a lot of leeway in that question.” (CYP/18, female, 18 years)

“I don’t think it might be that important.” (CYP/21, female, 15 years)

“It’s not particularly important” [laugh] (CYP/20, male, 18 years)

“Yeah it’s like if I saw that on a website as I was like scrolling through, I think I’d skim it but I wouldn’t like, it wouldn’t be the one that I’m there like, okay this is the most important question.” (CYP/19, female, 16 years)

“I think like sometimes, like especially in specific conditions really of trials, it’s kind of self-explanatory.” (CYP/18, female, 18 years)

“Red!”

“Yes, I’d say red” (CYP/18, female, 18 years)

“Yeah” (All)

#### Interaction of parent and CYP within joint interviews

For joint interviews, JMK encouraged participants to choose how they wanted to undertake the activity. Some parents watched and then added their thoughts after their child had completed the activity. This enabled parents’ views to be captured separately and contrasted with their child’s responses, without the views of either party dominating over the other. Parents sometimes talked to their child about what the topics on the cards meant, helping the child to decide where they wanted to place it. The activity offered the opportunity for JMK to ask questions about that topic as the card was placed. This process of reviewing after each card felt more natural than waiting until the end of the exercise and talking through the overall card placement. In some joint interviews with parents/carers and CYP, one participant would rank the card and then the other participant would say immediately where they would rank it and why. This enabled discussion and for participants to say if they disagreed on the ranking of a topic. A joint interview between a grandfather and his granddaughter, who was in hospital for treatment of a long-term health condition, showed how she felt comfortable to challenge the ranking that her grandfather initially gave:

“Do I have to take part?” [reading out card] (CYP/29, female, 12 years)

“I don’t think that’s important” (Grandfather, Parent/29)

“It’s up to you [taking part]. That’s very important, it’s your decision. And then this one, ‘what are the possible benefits of taking part?” [reading out card] (CYP/29, female, 12 years)

“Do you think that goes in the red or would you put that in the blue?” (Grandfather, Parent/29)

“Benefits are the good things about taking part. I don’t know…I think the red, you want to get something out of it don’t you.” (CYP/29, female, 12 years)

### Engagement and enjoyment

Participants laughed and talked to *each other* more during the exercise of ranking the topics on the target, than they did for the initial part of the interview or focus group discussion. This is illustrated by this conversation in a focus group:

“What will happen to the results of the study?” [reading out card] (CYP/20, male, 18 years)

“We like that” [all speaking]

*“We do”* (CYP/20, male, 18 years)

“That goes in the yellow. Everyone agreed?” (CYP/18, female, 18 years)

“Do I have to take part?” [reading card] (CYP/19, female, 16 years)

All: “Yeah that’s important. Yeah”

“I’m going to put that right in the middle. Does everyone agree?” (CYP/19, female, 16 years)

“Yeah. Yes.” (All)

“I’m really enjoying this!” [laughs] (CYP/18, female, 18 years)

The exercise also worked when interviewing individual participants, as the discussion continued between the participant and researcher. One CYP (9 years old) described as ‘very shy’ by his father, did not engage with the ranking exercise (and showed limited interest in the interview overall). However, all the other CYP were very animated with clapping and spontaneous chatting about the importance of each card. Younger CYP (10–13 year olds) particularly enjoyed reading aloud the card and then describing where this card should be placed on the target. Participants showed a lot of enthusiasm and animated positioning of cards on the target, with care taken to put cards in an exact position on the target.

Some CYP and parents, ranked *within* each category. For example, the ‘somewhat important’ category had two layers or circles of red (see Figure 1) and participants would sometimes say something was ‘inner red’ or ‘outer red’ which was not something the research team had anticipated ahead of the activities.

One CYP worked through the exercise independently and approached it as a ‘think-aloud’ task reading out the card and then verbalising his thoughts on what it meant to him and how he would rank it:

“What would happen if I want to stop being in the study [reading out card], is quite important. I don’t know. There would be things more important but I don’t think it should go in red, so I’ll put it in yellow and might move it at the end. Who is paying for the research?’ [reading out card] Honestly I don’t think that is too important to know, so I’ll pop that there. Who has checked the study is okay? [reading out card] Probably? You don’t want random people who you don’t understand what they’re doing or seeing what you’re doing so I’ll put that in yellow because if they actually did check it might have like things like your address and stuff but you don’t know who they really are and you don’t know what they’re going to do with that, so you need to know who it is. What will I have to do if I take part? [reading out card], is important. That can go in yellow as well. There’s a lot of yellows” (CYP/16, male, 10 years)

#### Professionals’ participation

Interestingly, professionals also enjoyed and engaged with the activity. The research team was initially unsure whether professionals would find the activity ‘too young’ but all professionals engaged with the exercise and ranked the topics, even more senior professionals. There was often a lot of laughter and chatting within their groups about who would take on the role of reading out the cards and who would place the cards on the target. When the ranking was undertaken in focus groups, if groups had four or more participants, participants divided themselves into smaller groups of two to four people. At the end of the ranking exercise, professional participants looked over and compared their rankings with other groups; with one professional remarking:

“They [the rankings] are quite similar, it’s surprising.” (Professional/1, female)

##### Use of cards to rank topics and suggesting additional topics

One CYP commented that the topics on the cards were ‘all valid’ to cover but individually they would ‘not have been able to recall and discuss all of these topics’ without having the cards to refer to. It also ensured that all topics were consistently covered with each participant so that we could get an overall view on the importance of each topic ahead of developing the websites. It was possible that the use of cards closed the discussion to only cover the information on the cards. However, allowing participants the option to offer any topics not covered on the cards at the end of the activity also elicited a few additional topics and their respective ranking. These topics included how taking part in a trial would affect their current care, whether they would be able to access medication after the trial, and how many participants were being recruited into the trial.

##### Researcher perspective of the activity

From the perspective of the interviewer, she felt the exercise facilitated discussion and also provided a record of the discussion and final decisions (through photographs of the exercise). Posing ‘why’ questions in interviews can be challenging, with participants feeling they have to justify their views. Having the shared visual focus of the cards made it easier to explore why participants ranked a particular card as they did – the focus was on the placing of card, not just the participant. This was particularly noticeable in the focus groups where the group would ask each other about why they felt a particular topic was important; sometimes a participant’s initial response changed after these types of discussions. Sometimes, in focus groups, a participant would quickly announce a ranking (important, not important) and then other participants would query this, giving an example of why they felt it should be ranked differently. The activity seemed to support participants, particularly CYP, to convey their views but also avoiding them being able to guess what the ‘expected response’ was. It also enabled CYP to place the cards and consider each in relation to other cards so that they could consider their placement and ranking throughout the exercise and also after discussion with other participants (for focus groups and joint interviews).

## Discussion

This paper describes the usefulness of an information ranking exercise that assisted engagement and discussion about the information CYP feel is important to know before taking part in a clinical trial. This topic was complex and for some, an unfamiliar, concept. By using cards with topics and asking participants to rank, we were able to compare and contrast the views of different participant groups (CYP; parents/carers; professionals). The method used a school-like discussion technique that participants found acceptable, and interestingly, it worked well with professionals as well as CYP, demonstrating the applicability of this type of activity for a range of participants (Niemi et al., 2015) and that thinking creatively when we communicate has benefits for a range of audiences.

This study adds to the growing literature describing the use of CYP-friendly participatory research methods (Colucci, 2007; Flanagan et al., 2015; Gibson et al., 2010; Niemi et al., 2015). Specifically, it demonstrates the benefits of using participatory visual ranking activities with CYP in research, a relatively simple, inexpensive technique, which provided a method of engaging particularly CYP in a topic that may seem difficult. Importantly the activity helps to avoid participants thinking the researchers want a ‘particular’, ‘right’ or ‘correct’ answer as the activity provides a shared focus other than the participants’ responses. By using cards that are movable, participants have time to consider where they feel the card is best placed; the activity also allows discussion between participants. In value-based research there is a risk that participants will try to second-guess what the researcher is expecting or wanting as a response and this ranking helped to reduce that risk. We also feel that using visual methods with cards in qualitative research would be useful in other activities where ranking may not be the focus, to assist discussion of topics or ideas.

Whilst there is limited literature on the experience of using interactive and novel ranking methods to engage CYP in research, there are some studies demonstrating benefits of using activities within qualitative research. Many explore the use of visual and spatial methods such as drawings and photography to support and engage CYP in discussion (Crilly et al., 2006; Lobinger and Brantner, 2020; White et al., 2010; Woolner et al., 2010). Others have described the benefits of methods to specifically access views of CYP with impaired communication or cognition and thereby promote inclusivity (Boggis, 2011; Morris, 2003), the use of visual methods and techniques within education setting (Wall et al., 2013) and the contribution of visual methods to inclusive education (Prosser and Loxley, 2007). The value of using diagrams and graphs to complement conventional interview structures and support participants has also been reported (Crilly et al., 2006). In line with our observations, Fängström et al. (2017) has proposed that the use of interactive activities within interviews shifts the focus from the person to the activity (Fängström et al., 2017) so reducing the social demands of the interview and stress associated with traditional interviewing formats, where participants may worry about whether they have answered the question correctly (Fängström et al., 2017).

### Strengths and limitations

While we report here that the ranking method was acceptable and accessible to participants, we acknowledge the possibility that the method constrained discussion to the topics on the cards. Nevertheless, some participants suggested other pieces of information at the end of the exercise. A further potential limitation is that we combined data from individual interviews, joint interviews and focus group in the analysis. However, we did this to be pragmatic and accommodate participants’ preferences for participating in the study and we note that the literature shows the benefits of combining both interviews and focus groups by increasing richness of the data (Lambert and Loiselle, 2008; Lewis and McNaughton Nicholls, 2017; Michel, 2011). Findings from the ranking exercise successfully informed the shape and structure of the multimedia websites that were developed within the TRECA study. The use of an interactive activity was planned from the beginning of the study for interviews with all participant groups (Martin-Kerry et al., 2017). Future research on the various tools such as ranking to engage CYP would be worthwhile.

### Implications for practice

This visual method of ranking is helpful in engaging CYP as well as adults. It enables discussion of content in semi-structured interviews and focus groups about topics which may otherwise be difficult for CYP to discuss. Importantly, the ranking exercise is simple and inexpensive, it does not require researchers to have prior training and uses materials that are readily available. We recommend other researchers consider using this ranking method within future qualitative work with CYP.

## Conclusions

There is limited information in the peer-reviewed literature about the practicalities of using interactive information ranking exercises for engaging CYP in health research. This paper describes a way to actively engage with participants of different ages and from different stakeholder groups in a qualitative study where ranking of information was needed. In the TRECA study it formed an invaluable stage in supporting the co-design of the multimedia websites. By using this method, we elicited insightful discussion about what information needed to be most prominent within the multimedia websites.

## Supplemental Material

Supplemental Material - Engaging children, young people, parents and health professionals in interviews: Using an interactive ranking exercise within the co-design of multimedia websites Supplemental Material for Engaging children, young people, parents and health professionals in interviews: Using an interactive ranking exercise within the co-design of multimedia websites by Jacqueline Martin-Kerry, Steven Higgins, Peter Knapp, Kristin Liabo and Bridget Young in Journal of Child Health Care

## References

[bibr4-13674935221109684] Boggis A (2011) Deafening silences: researching with inarticulate children. Disability Studies Quarterly 31(4). DOI: 10.18061/dsq.v31i4.1710

[bibr5-13674935221109684] Braun V Clarke V (2006) Using thematic analysis in psychology. Qualitative Research in Psychology 3: 77–101.

[bibr6-13674935221109684] Braun V Clarke V (2014) What can “thematic analysis” offer health and wellbeing researchers? International Journal of Qualitative Studies in Health and Well-Being 9: 26152. DOI: 10.3402/qhw.v9.26152 PMC4201665 25326092

[bibr7-13674935221109684] Caldwell P Dans L de Vries M , et al. (2012) Standard 1: consent and recruitment. Pediatrics 129: S118–S123. 22661757 10.1542/peds.2012-0055D

[bibr8-13674935221109684] Caldwell PH Murphy SB Butow PN , et al. (2004) Clinical trials in children. Lancet 364: 803–811. 15337409 10.1016/S0140-6736(04)16942-0

[bibr9-13674935221109684] Christian BJ Pearce PF Roberson AJ , et al. (2010) It’s a small, small world: data collection strategies for research with children and adolescents. Journal of Pediatric Nursing 25: 202–214. 20430281 10.1016/j.pedn.2009.01.003

[bibr10-13674935221109684] Clark A (2005) Listening to and involving young children: a review of research and practice. Early Child Development and Care 175: 489–505.

[bibr11-13674935221109684] Colucci E (2007) “Focus groups can be fun”: the use of activity-oriented questions in focus group discussions. Qualitative Health Research 17: 1422–1433. 18000081 10.1177/1049732307308129

[bibr12-13674935221109684] Crane S Broome ME (2017) Understanding ethical issues of research participation from the perspective of participating children and adolescents: a systematic review. Worldviews of Evidence Based Nursing 14: 200–209. 10.1111/wvn.12209 PMC5724520 28207982

[bibr13-13674935221109684] Crilly N Blackwell AF Clarkson PJ (2006) Graphic elicitation: using research diagrams as interview stimuli. Qualitative Research 6: 341–366.

[bibr14-13674935221109684] Curtin C (2001) Eliciting children’s voices in qualitative research. American Journal of Occupational Therapy 55: 295–302. 10.5014/ajot.55.3.295 11723970

[bibr15-13674935221109684] Dunhill A Elliott B Shaw A (2009) Effective communication and engagement with children and young people, their families and carers. Exeter: Learning Matters.

[bibr16-13674935221109684] Fängström K Salari R Eriksson M , et al. (2017) The computer-assisted interview in my shoes can benefit shy preschool children’s communication. Plos One 12: e0182978–e0182978. 28813534 10.1371/journal.pone.0182978 PMC5557539

[bibr17-13674935221109684] Fargas-Malet M McSherry D Larkin E , et al. (2010) research with children: methodological issues and innovative techniques. Journal of Early Childhood Research 8: 175–192.

[bibr18-13674935221109684] Flanagan SM Greenfield S Coad J , et al. (2015) An exploration of the data collection methods utilised with children, teenagers and young people (CTYPs). BMC Research Notes 8: 61. 25888787 10.1186/s13104-015-1018-y PMC4359510

[bibr19-13674935221109684] Ford K Sankey J Crisp J (2007) Development of children’s assent documents using a child-centred approach. Journal of Child Health Care 11: 19–28. 17287221 10.1177/1367493507073058

[bibr20-13674935221109684] Gibson F Aldiss S Horstman M , et al. (2010) Children and young people’s experiences of cancer care: a qualitative research study using participatory methods. International Journal of Nursing Studies 47: 1397–1407. 20430388 10.1016/j.ijnurstu.2010.03.019

[bibr21-13674935221109684] Joseph PD Craig JC Caldwell PH (2015) Clinical trials in children. British Journal of Clinical Pharmacology 79: 357–369. 24325152 10.1111/bcp.12305 PMC4345947

[bibr22-13674935221109684] Kirk S (2007) Methodological and ethical issues in conducting qualitative research with children and young people: a literature review. International Journal of Nursing Studies 44: 1250–1260. 17027985 10.1016/j.ijnurstu.2006.08.015

[bibr23-13674935221109684] Kirkby HM Calvert M Draper H , et al. (2012) What potential research participants want to know about research: a systematic review. BMJ Open 2: e000509. 10.1136/bmjopen-2011-000509 PMC3367142 22649171

[bibr24-13674935221109684] Klassen TP Hartling L Craig JC , et al. (2008) Children are not just small adults: the urgent need for high-quality trial evidence in children. Plos Medicine 5: e172. 18700813 10.1371/journal.pmed.0050172 PMC2504487

[bibr25-13674935221109684] La Pelle N (2004) Simplifying qualitative data analysis using general purpose software tools. Field Methods 16: 108–185.

[bibr26-13674935221109684] Lambert SD Loiselle CG (2008) Combining individual interviews and focus groups to enhance data richness. Journal of Advance Nursing 62: 228–237. 10.1111/j.1365-2648.2007.04559.x 18394035

[bibr27-13674935221109684] Lewis J McNaughton Nicholls C (2017) Design issues. In: Ritchie J Lewis J McNaughton C (eds.) Qualitative Research Practice. London: Sage Publishing.

[bibr28-13674935221109684] Lobinger K Brantner C (2020) Picture. In: Pauwels L Mannay D (eds) The SAGE Hanbook of Visual Research Methods. 2nd revised and expanded edition. London: Sage Publishing.

[bibr100-13674935221109684] Martin-Kerry J Bower P Young B , et al. (2017) Developing and evaluating multimedia information resources to improve engagement of children, adolescents, and their parents with trials (TRECA study): Study protocol for a series of linked randomised controlled trials. Trials 18(1): 265. 28595613 10.1186/s13063-017-1962-z PMC5465557

[bibr101-13674935221109684] Martin-Kerry JM Knapp P Atkin K , et al. (2019) Supporting children and young people when making decisions about joining clinical trials: qualitative study to inform multimedia website development. BMJ Open 9(1): e023984. 10.1136/bmjopen-2018-023984 PMC6340013 30782720

[bibr29-13674935221109684] Michel L (2011) Combining focus groups and interviews: telling how it is; telling how it feels. In: Barbour RS Kitzinger J (eds) Developing Focus Group Research. London: SAGE Publications.

[bibr30-13674935221109684] Morgan M Gibbs S Maxwell K , et al. (2002) Hearing children's voices: methodological issues in conducting focus groups with children aged 7-11 years. Qualitative Research 2: 5–20.

[bibr31-13674935221109684] Morris J (2003) Including all children: finding out about the experiences of children with communication and/or cognitive impairments. Children & Society 17: 337–348.

[bibr32-13674935221109684] Niemi R Kumpulainen K Lipponen L (2015) Pupils as active participants: diamond ranking as a tool to investigate pupils’ experiences of classroom practices. European Educational Research Journal 14: 138–150.

[bibr33-13674935221109684] Nuffield Council on Bioethics (2015) Children and Clinical Research: Ethical Issues. London: Nuffield Council on Bioethics.

[bibr34-13674935221109684] O’Connell R (2013) The use of visual methods with children in a mixed methods study of family food practices. International Journal of Social Research Methodology 16: 31–46.

[bibr35-13674935221109684] Patel ZS Jensen SE Lai JS (2016) Considerations for conducting qualitative research with pediatric patients for the purpose of PRO development. Quality of Life Research 25: 2193–2199. 26941216 10.1007/s11136-016-1256-z

[bibr36-13674935221109684] Prosser J Loxley A (2007) Enhancing the contribution of visual methods to inclusive education. Journal of Research in Special Educational Needs 7: 55–68.

[bibr37-13674935221109684] Punch S (2002) Interviewing strategies with young people: the ‘secret box’, stimulus material and task-based activities. Children & Society 16: 45–56.

[bibr38-13674935221109684] Rockett M Percival S (2002) Thinking for Learning. Stafford: Network Educational Press.

[bibr102-13674935221109684] Sheridan R Martin-Kerry J Watt I , et al. (2019) User testing digital, multimedia information to inform children, adolescents and their parents about healthcare trials. J Child Health Care 23(3): 468–482. DOI: 10.1177/1367493518807325. 30384772

[bibr39-13674935221109684] Thomas N O’Kane C (1998) The ethics of participatory research with children. Children & Society 12: 336–348.

[bibr40-13674935221109684] Tishler CL Reiss NS (2011) Pediatric drug-trial recruitment: enticement without coercion. Pediatrics 127: 949–954. 21464193 10.1542/peds.2010-2585

[bibr41-13674935221109684] Vindrola-Padros C Martins A Coyne I , et al. (2016) From informed consent to dissemination: using participatory visual methods with young people with long-term conditions at different stages of research. Global Public Health 11: 636–650. 27219895 10.1080/17441692.2016.1165718

[bibr42-13674935221109684] Wall K Higgins S (2006) Facilitating metacognitive talk: a research and learning tool. International Journal of Research & Method in Education 29: 39–53.

[bibr43-13674935221109684] Wall K Higgins S Hall E , et al. (2013) ‘That’s not quite the way we see it’: the epistemological challenge of visual data. International Journal of Research & Method in Education 36: 3–22.

[bibr44-13674935221109684] White A Bushin N Carpena-Méndez F , et al. (2010) Using visual methodologies to explore contemporary Irish childhoods. Qualitative Research 10: 143–158.

[bibr45-13674935221109684] Woolner P Clark J Hall E , et al. (2010) Pictures are necessary but not sufficient : using a range of visual methods to engage users about school design. Learning Environments Research 13: 1–22.

