# Supplementary material for: Engaging children, young people, parents and health professionals in interviews: Using an interactive ranking exercise within the co-design of multimedia websites
Source: J Child Health Care. 2022 Jun 24;28(1):181–95. doi: 10.1177/13674935221109684 (PMC10882948; doi:10.1177/13674935221109684)
Supplement: Supplemental Material - Engaging children, young people, parents and health professionals in interviews: Using an interactive ranking exercise within the co-design of multimedia websites [file sj-pdf-1-chc-10.1177_13674935221109684.pdf]

Supplementary file 1: Participant characteristics

Children and Young people			
**Participant ID**	**Gender**	**Age (years)**	**Experience of being approached about a children's trial**
CYP/16	Male	10	Yes
CYP/18	Female	18	Yes
CYP/19	Female	16	No
CYP/20	Male	18	No
CYP/21	Female	15	No
CYP/23	Female	16	Yes
CYP/25	Male	9	Yes
CYP/29	Female	12	No
CYP/31	Female	19	Yes
CYP/35	Female	16	No
CYP/39	Female	14	No
CYP/43	Male	9	Yes
CYP/44	Male	6	No
CYP/47	Male	6	No
CYP/49	Male	7	Yes
CYP/51	Female	15	Yes
CYP/53	Female	16	No
CYP/55	Female	6	No
CYP/57	Female	6	Yes
CYP/59	Male	13	No
CYP/62	Female	8	Yes
Parents/carers			
**Participant ID**	**Gender**	**Experience of being approached about a children's trial**	
Parent/17	Female	Yes	
Parent/22	Female	Yes	
Parent/24	Female	Yes	
Parent/26	Female	Yes	
Parent/27	Male	Yes	
Parent/28	Male	No	
Parent/30	Female	No	

Parent/32	Female	No
Parent/33	Male	No
Parent/34	Female	No
Parent/36	Female	No
Parent/37	Female	Yes
Parent/38	Female	No
Parent/41	Female	Yes
Parent/42	Male	Yes
Parent/46	Female	Yes
Parent/48	Male	Yes
Parent/50	Female	Yes
Parent/52	Male	No
Parent/54	Female	No
Parent/56	Female	No
Parent/58	Male	Yes
Parent/60	Male	No
Parent/61	Female	Yes
	Professionals	
**Participant ID**	**Gender**	**Current role**
Professional/1	Female	Paediatric rheumatology consultant
Professional/2	Female	Paediatric rheumatology consultant
Professional/3	Female	Paediatric renal consultant
Professional/4	Female	Paediatric intensive care nurse
Professional/5	Female	Operations manager
Professional/6	Female	Paediatric research nurse
Professional/7	Female	Research governance (previous researcher)
Professional/8	Female	Research pharmacist
Professional/9	Female	Research nurse
Professional/10	Female	Research nurse
Professional/11	Male	Research nurse
Professional/12	Female	Research nurse
Professional/13	Female	Data manager (involved in recruitment)
Professional/14	Male	Paediatric respiratory consultant
Professional/15	Male	Paediatric respiratory consultant

Professional/40	Female	Play specialist
Professional/45	Male	Paediatric pharmacist
